# Constitutive Serotonin Tone Modulates Molecular and Behavioral Response to Chronic Fluoxetine Treatment: A Study on Genetic Rat Model

**DOI:** 10.3389/fpsyt.2021.741222

**Published:** 2021-10-01

**Authors:** Maja Kesić, Gordana Mokrović, Ante Tvrdeić, Branko Miše, Jasminka Štefulj, Lipa Čičin-Šain

**Affiliations:** ^1^Laboratory for Neurochemistry and Molecular Neurobiology, Department of Molecular Biology, Ruđer Bošković Institute, Zagreb, Croatia; ^2^Department of Pharmacology, School of Medicine, University of Zagreb, Zagreb, Croatia; ^3^University Hospital for Infectious Diseases, Zagreb, Croatia

**Keywords:** 5-HT, serotonin transporter, animal model, SSRI, anxiety, depression

## Abstract

Selective serotonin reuptake inhibitors (SSRIs) are the most commonly prescribed medications for the treatment of mood disorders. Yet, individual response to SSRIs is highly variable, with only a portion of patients showing the desired therapeutic effect. To better understand the molecular basis underlying individual variability in response to SSRIs, here we comparatively studied behavioral and molecular consequences of chronic treatment with fluoxetine, a widely used SSRI, in two sublines of rats with constitutionally different serotonin (5HT) homeostasis: the high-5HT and low-5HT sublines. Platelet 5HT levels, a recognized indicator of SSRI efficacy, were decreased by fluoxetine treatment in both 5HT-sublines. On the other hand, biologically active plasma 5HT levels were reduced only in high-5HT rats. The anxiolytic effect of fluoxetine was also evident only in high-5HT rats, as supported by spatio-temporal and ethological behavioral measures in the elevated plus maze (EPM) test and exploratory behavior measures in the open field (OF) test. None of the behavioral EPM or OF measures were significantly altered by fluoxetine treatment in low-5HT rats. Unexpectedly, 5HT levels in cerebral cortices tended to be reduced only in low-5HT rats. Moreover, the effects of fluoxetine on cortical expression levels of 5HT-related proteins were also present only in low-5HT rats, with serotonin transporter (*5HTT*) and serotonin receptor type 1a (*Htr1a*) being down-regulated, while serotonin receptor type 4 (*Htr4*) was up-regulated by fluoxetine treatment. The obtained results support a role of individual 5HT tone as an important influencing factor on the biological actions of SSRI antidepressants.

## Introduction

Fluoxetine is one of the most frequently prescribed medications for the treatment of depression and other behavioral symptoms associated with a deficiency in serotonin (5-hydroxytryptamine, 5HT) neurotransmission (1). It is the oldest member of the selective serotonin reuptake inhibitors (SSRIs), drugs that specifically block activity of the serotonin transporter (5HTT, SERT), a high-affinity 5HT carrier responsible for reuptake of 5HT from the synaptic cleft. A therapeutic response to SSRIs occurs only after several weeks of treatment, suggesting that rapid blockage of 5HT reuptake, followed by an immediate increase in synaptic 5HT levels, is not sufficient for the therapeutic effect. Thus, the therapeutic action of chronic SSRI treatment is thought to result from more complex adaptive changes in the brain serotonergic system. A primary adaptive mechanism involves desensitization of the presynaptic 5HT1A autoreceptors, but roles of other subtypes of 5HT receptors have also been proposed [for a review see (2)].

Despite the widespread use of SSRIs in the treatment of depression and other mood disorders, their therapeutic success shows marked individual variability. An adequate response to treatment is achieved in only up to 50% of patients with major depression and some patients experience an initial worsening of symptoms. In recent decades, much effort has been devoted to the search for molecular basis and markers of inter-individual differences in therapeutic response to SSRIs. One of the most promising lines of research has focused on functional genetic variants in the human serotonin transporter gene (3). Thus, a long (L) variant of the upstream regulatory region of the 5HTT gene, conferring higher 5HTT activity compared to the short (S) variant, has been associated with better response to SSRI treatment in Caucasian populations (4, 5). Additionally, lower DNA methylation levels in the 5HTT promoter region, associated with higher gene expression levels (6), were also found to predict better response to SSRI treatment (7). The role of 5HTT in modulating behavioral and molecular responses to SSRIs has also been addressed by experimental studies employing various animal models, such as rodents with targeted deletion or overexpression of the *5HTT* gene (8, 9) of mouse and rat strains with innate differences in 5HTT activity (10, 11). These studies have clearly established 5HTT as a primary target of SSRIs, but the molecular mechanisms underlying a link between constitutive 5HTT activity and therapeutic SSRI efficacy are not well-understood.

By selective breeding of Wistar rats for extreme values of platelet 5HT level, we have developed an animal model named the Wistar Zagreb-5HT (WZ-5HT) rat, comprising of two sublines of animals with inherited differences in blood 5HT levels (12). Subsequent studies have shown that animals from our high-5HT and low-5HT sublines also display constitutionally altered (i.e., up-regulated or down-regulated, respectively) platelet 5HTT activity (13). Since 5HTT in platelets and neurons is encoded by the same gene, it was expected that rats from WZ-5HT sublines would also differ in brain serotonergic homeostasis. Indeed, neurochemical, behavioral and neuropharmacological studies provided evidence for differential functionality of central 5HT systems between 5HT-sublines. Thus, animals from the high-5HT subline, compared to the low**-**5HT subline, show higher 5HT turnover and ^3^H-citalopram binding in several brain regions as well as higher KCl- and citalopram-induced elevation in extracellular 5HT in hippocampal tissue (14). A number of functional traits modulated by central and/or peripheral 5HT signaling differ between 5HT sublines (15–20) and the pattern of changes is consistent with relatively higher whole-body 5HT activity in high-5HT as compared to low-5HT animals.

To better understand the molecular basis underlying inter-individual variability in response to SSRI pharmacotherapy, here we comparatively studied behavioral and molecular consequences of chronic fluoxetine treatment in rats from high-5HT and low-5HT subline. In previous studies, we have shown that animals from the respective sublines display different performance in an open field (OF) and elevated plus maze (EPM) tests (15, 16), a two well-known paradigms for assessing functioning of brain regions involved in anxiety (21). Hence, here we investigated fluoxetine-induced changes in the behavior of WZ-5HT rats in OF/EPM tests. In addition, we assessed the expression levels of several key genes regulating 5HT neurotransmission in the frontal cortex, an important brain region involved in antidepressant actions, and examined 5HT levels in both the frontal cortex and the periphery (blood platelets and plasma compartments).

## Materials and Methods

### Animals

Studies were performed on high-5HT and low-5HT sublines of the WZ-5HT rats, developed at the Ruder Bošković Institute, Zagreb, Croatia, by selective breeding of Wistar rats toward extremes of their platelet serotonin levels (PSL) and velocity of platelet serotonin uptake (PSU). Development of the WZ-5HT rats has been described elsewhere (12, 13). Briefly, at the age of 5–6 weeks, PSL and PSU were determined in all offspring of each breeding generation and the males and females displaying the extreme values of both parameters were mated to start a next generation. After 5–6 generations of selective breeding, the divergence of mean values of platelet 5HT parameters between 5HT sublines stabilizes at about 70% (low-5HT subline) and 150% (high-5HT subline) of the initial (unselected) population (12). Selective breeding was restarted *ab ovo* for several times during the past decades with essentially the same final divergence of sublines, confirming reproducibility of the directed selection process. In the present study 13–14 weeks old male WZ-5HT rats were used. They were housed 3 per cage with free access to commercial rat chow (4RF18 GLP, Mucedola Srl, Settimo Milanese, Italy) and tap water, and were kept under controlled conditions (23 ± 2°C, 55 ± 10% humidity, 12 h light/12 h dark). All experiments were approved by the institutional and national (Ministry of Agriculture, Republic of Croatia) ethical committees and were conducted in accordance with the EU directive on the protection of animals used in scientific purposes (2010/63/EU) and ILAR Guide for the Care and Use of Laboratory Animals.

### Treatment of Animals and the Timeline of Analyses

**Figure 1:**
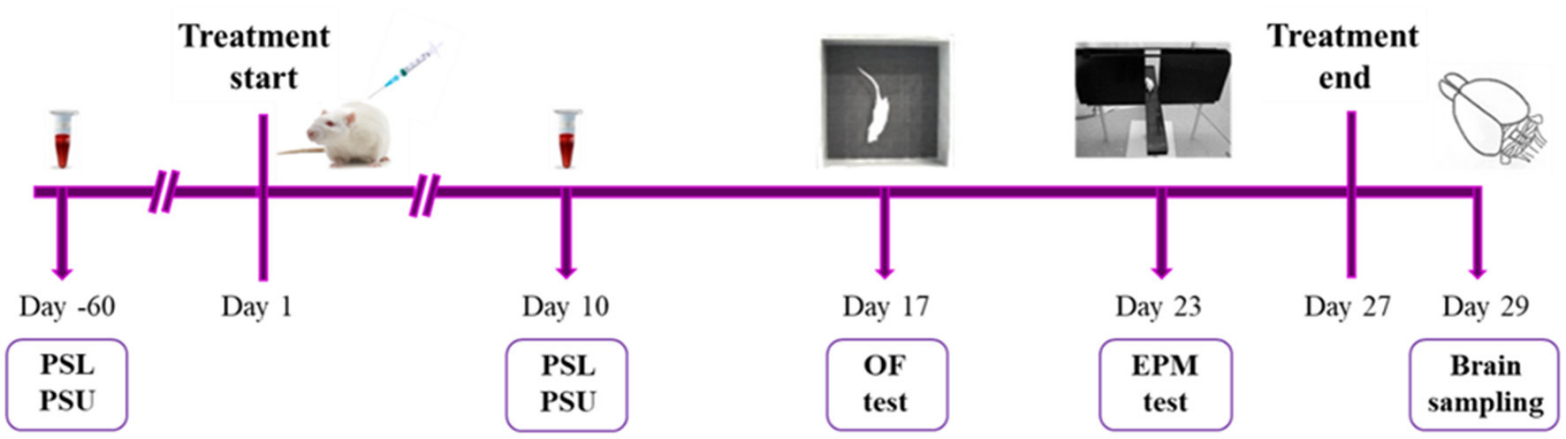
A scheme illustrating experimental design and time course of analyses. At the age of 5–6 weeks (60 days before the start of the treatment), animals from high-5HT and low-5HT subline were subjected to blood sampling for verification of platelet serotonin level (PSL) and platelet serotonin uptake (PSU). At the age of 13–14 weeks, rats from both sublines were randomly divided into two groups and for the following 27 days were daily treated with i.p. injections of either vehicle (*n* = 8 per subline) or fluoxetine (6 mg/kg; *n* = 17 per subline). On day 10 of treatment, all animals receiving vehicle and subset of animals receiving fluoxetine (*n* = 8 per subline) were subjected to blood sampling for PSL and PSU measurements. On days 17 and 23 of treatment, all animals underwent behavioral assessment in open field (OF) and elevated plus maze (EPM) test, respectively. 48 h after the last (27th) injection of vehicle or fluoxetine, animals were sacrificed, and cortical brain tissue samples were taken for 5HT level measurements and gene expression analyses.

Animals from high-5HT and low-5HT sublines were treated with intraperitoneal (i.p.) injection of vehicle (0.9% saline; *n* = 8 per subline) or fluoxetine hydrochloride (a gift from Lilly Research Laboratories, Indianapolis, IN, USA; *n* = 17 per subline) for 27 days. Because of the expected greater interindividual variability in the behavioral response of drug-treated animals compared to vehicle-treated animals, a larger number of fluoxetine-treated animals per subline was included. The daily dose of fluoxetine (6 mg/kg of body weight) was chosen based on literature data. In rodents, chronic fluoxetine treatment is usually administered at a dose of 5–10 mg/kg/day for 2–4 weeks (22–25). We used a moderate daily dose of 6 mg/kg, which mimics well the therapeutic doses of the drug in human patients (see Discussion for more details), and chose a longer treatment period (27 days) to allow sufficient time interval between blood collection and behavioral testing. The drug solution in 0.9% saline was freshly prepared each day and administered at a dosage of 0.5 ml/100 g of body weight. Blood sampling and behavioral experiments were started 24 h after a pre-determined number of injections, while brain samples for neurochemical and molecular analyses were obtained after 48 h drug wash-out period. The timing for measuring blood 5HT parameters was based on our previous results with fluvoxamine (18), which showed that platelet 5HT levels in the treated group remained stably low from day 4 of treatment onwards, so a similar time-response could be expected in the present study with fluoxetine. All drug treatments were done between 9 and 10 a.m. except on the days of blood sampling and behavioral testing, when the drug was given immediately after completion of the respective procedures. The time-line of biochemical, behavioral and molecular biological analyses is shown in Figure 1.

### Blood Sampling and 5HT-Related Biochemical Measurements

Protocols for repetitive rat blood sampling, preparation of platelet-rich plasma (PRP) and determination of PSL and PSU have been described elsewhere (26, 27). In short, venous blood was withdrawn by jugular venipuncture and PRP was isolated by centrifugation (1,050×g for 25 s). PSU was determined radiometrically by incubating aliquots of PRP with 0.5 μM ^14^C-5HT followed by rapid cooling and vacuum filtration. At this 5HT concentration the velocity of PSU is ~70% of the maximal velocity (V_max_) (13). For measurement of PSL, platelets were pelleted, homogenized by sonication and 5HT content was quantified spectrophotofluorimetrically and related to platelet protein. Platelet-free plasma (PFP) was obtained by a two-step centrifugation: anticoagulated blood was centrifuged (1,050×g for 4 min), the upper layer of supernatant plasma was carefully collected, recentrifuged for 30 min and collected PFP was stored at −80°C for further testing. 5HT levels in plasma and brain tissue were analyzed by enzyme-linked immunosorbent assay (ELISA; Demeditec Diagnostics GmbH, Kiel, Germany and IBL International GmbH, Hamburg, Germany, respectively) according protocol supplied by the manufacturer.

### Behavioral Testing

Behavioral performance of the animals was assessed by the open field (OF) and elevated-plus maze (EPM) tests. Animals were naive to the test situations and were brought into the test room 1 h before testing. The activity of each rat during tests was recorded by web camera fixed above the behavioral apparatus and connected to computer outside the test room. Videos were analyzed using the ANY-maze video tracking system (Stoelting Co, Wood Dale, IL, USA). Behavioral apparatuses were thoroughly cleaned with a 70% ethanol after each test session to remove any odor residue. Rats from high-5HT and low-5HT sublines were always tested alternately.

#### Open Field Test

The OF test was performed in a rectangular arena (60 × 60 × 40 cm) made of white painted wood and virtually divided into border (12.5 cm from the edge) and central zone. Animals were placed, facing the wall, in the brightly illuminated arena (300 lux, light bulb 90 cm above the arena) and allowed to explore it for 5 min. Behavioral parameters recorded were: number of entries and time spent in central and border zones (measuring anxiety/exploration), distance traveled and mean speed (ambulation), frequency and time spent in rearing (exploration/locomotion), and grooming (displacement response) activity. Latencies to the first onset of activity (entry into central zone and rearing and grooming in the border zone) were also recorded. Rearing was defined as standing on the hind limbs and grooming included cleaning any part of the body surface with the tongue, teeth and/or forepaws.

The anxiolytic effect was assessed by both conventional (increased number of entries and time spent in the central zone and decreased time spent in the border zone) and non-conventional (increased frequency and time spent in rearing in the central zone and increased grooming in the whole apparatus) behavioral measures.

#### Elevated Plus Maze Test

The EPM test was performed in an apparatus consisting of two sets of opposing arms: two open (50 × 10 cm) and two enclosed (50 × 10 cm, surrounded by 40 cm high walls) arms made of black plexiglas and elevated 1 m above the floor. The central platform at the junction of the four arms was 10 × 10 cm. The illumination (light bulb 90 cm above the maze) was 125 lux in the open arms and 60 lux in the closed arms. Animals were placed on the central platform, with a head facing an open arm and allowed to explore the maze for 5 min. The following behavioral parameters were analyzed: number of entries and time spent in open and enclosed arms and on the central platform (anxiety/exploration), distance traveled in the enclosed arms and during the whole test and average speed during the test (ambulation), frequency and time devoted to rearing in enclosed arms (exploration/locomotion), as well as head dip number, duration and latencies to onset in both, open arms, and on the central platform (exploration/risk assessment). Entries were counted if minimum of 85% of the animal area was in the arm zone. Head-dipping was defined as exploratory leaning to the floor from the open arms and central square. The collected data were further used to calculate derived behavioral measures: % entries open and % time open (based on total entries and total test duration), the ratio between open time and closed time and the index of open arm avoidance calculated as follows: 100–[(% time on open arms + % entries to open arms)/2] (28).

As in the OF test, anxiolytic effect was assessed by both conventional (increased number of entries and time in open arms, increased % of time and % of entries in open arms) and non-conventional (increased head dip number and time in open arms) behavioral measures. Increased ratio of time in open to closed arms and decreased index of open arm avoidance were also considered anxiolytic markers (28). In both tests, measures of locomotor activity were not considered indicators of anxiolytic but of sedative/excitatory effect of fluoxetine.

### Brain Tissue Sampling

Brains were removed from the skull, rapidly cooled on dry-ice powder for 10 s, and then placed with ventral side facing the cold metal plate for dissection. Manual coronal sections were made with razor blade and cortex tissues, containing the pre-frontal cortices, were separated. Tissue samples for gene expression analyses were rapidly frozen in liquid nitrogen and, prior to storage at −80°C, homogenized in a denaturing solution containing 4 M guanidine thiocyanate (Sigma-Aldrich, Merck KGaA, Darmstadt, Germany). Cortical tissue samples for 5HT determinations were directly stored at −80°C until further processing.

### Gene Expression Analyses

Total RNA from ~60 mg of cortical tissue was isolated by standard acid guanidinium-phenol-chlorophorm extraction and additionally purified by RNeasy Mini Kit (Qiagen, Germantown, MD, USA) according to the manufacturer's protocol with optional on-column DNA digestion step. Concentration and purity of isolated RNAs were assessed by spectrophotometry (NanoDrop, ND-1000, Thermo Fisher Scientific, Waltham, MA, USA); A_260_/A_280_ ratios ranged between 2.04 and 2.09. Aliquots of RNA were subjected to agarose gel electrophoresis to check integrity; all samples showed sharp 28S and 18S bands at ratio of ~2:1. RNA samples were stored at −80°C until further processing. Expression levels of genes encoding the 5HT transporter (*5HTT*), 5HT receptor subtypes 1A (*Htr1a*), 2A (*Htr2a*), and 4 (*Htr4*) were analyzed using the reverse transcription (RT) - quantitative PCR (qPCR) based on SyberGreen detection chemistry. First strand cDNA was synthesized from 2 μg of each RNA in a final volume of 20 μl using the High Capacity RNA to cDNA Synthesis Kit (Life Technologies, Thermo Fisher Scientific, Waltham, MA, USA) according to the manufacturer's protocol. Control reactions lacking reverse transcriptase (no-RT) were prepared to test for contamination with genomic DNA. cDNAs were diluted to concentration of 10 ng/μl and stored in small aliquots at −20°C. Sequences of primers used in qPCR are listed in Supplementary Table 1. qPCR assays were prepared using the SyberGreen Master Mix and run on a 7300 Real Time PCR System (both from Applied Biosystems, Thermo Fisher Scientific, Waltham, MA, USA) according to the manufacturer's recommendations. Each qPCR plate included a 5-point standard curve with 2-fold serial dilution and all reactions were performed in triplicates. The quantification cycles (C_q_) in no-RT controls were either undefined or more than 15 cycles higher than that obtained from the respective cDNA samples. Relative expression levels were determined using a relative standard curve method, and the mean relative expression level of two reference genes, glyceraldehyde-3-phosphate dehydrogenase (*Gapdh*) and actin beta (*Actb*), was used for normalization of expression levels (29).

### Statistical Analyses

Data were analyzed using GraphPad Prism, version 6.00 (GraphPad Software, San Diego, CA, USA). Normality of the data distribution was tested by D'Agostino-Pearson omnibus test and the presence of outliers by Grubb's test. Experimental groups were compared by one-way analysis of variance (1w-ANOVA) when data were normally distributed, or by Kruskal-Wallis (KW) test if data deviated from normal distribution. *Post-hoc* analyses were performed using Fisher's least significant difference (LSD) test (following 1w-ANOVA) or Dunn's test (following KW test). Results are expressed as means ± standard deviation (SD) or medians with interquartile range (IQR), as appropriate. Differences were considered statistically significant if *p* < 0.05. *p*-values between 0.05 and 0.1 are shown numerically in graphs.

## Results

### Blood 5HT Parameters

**Figure 2:**
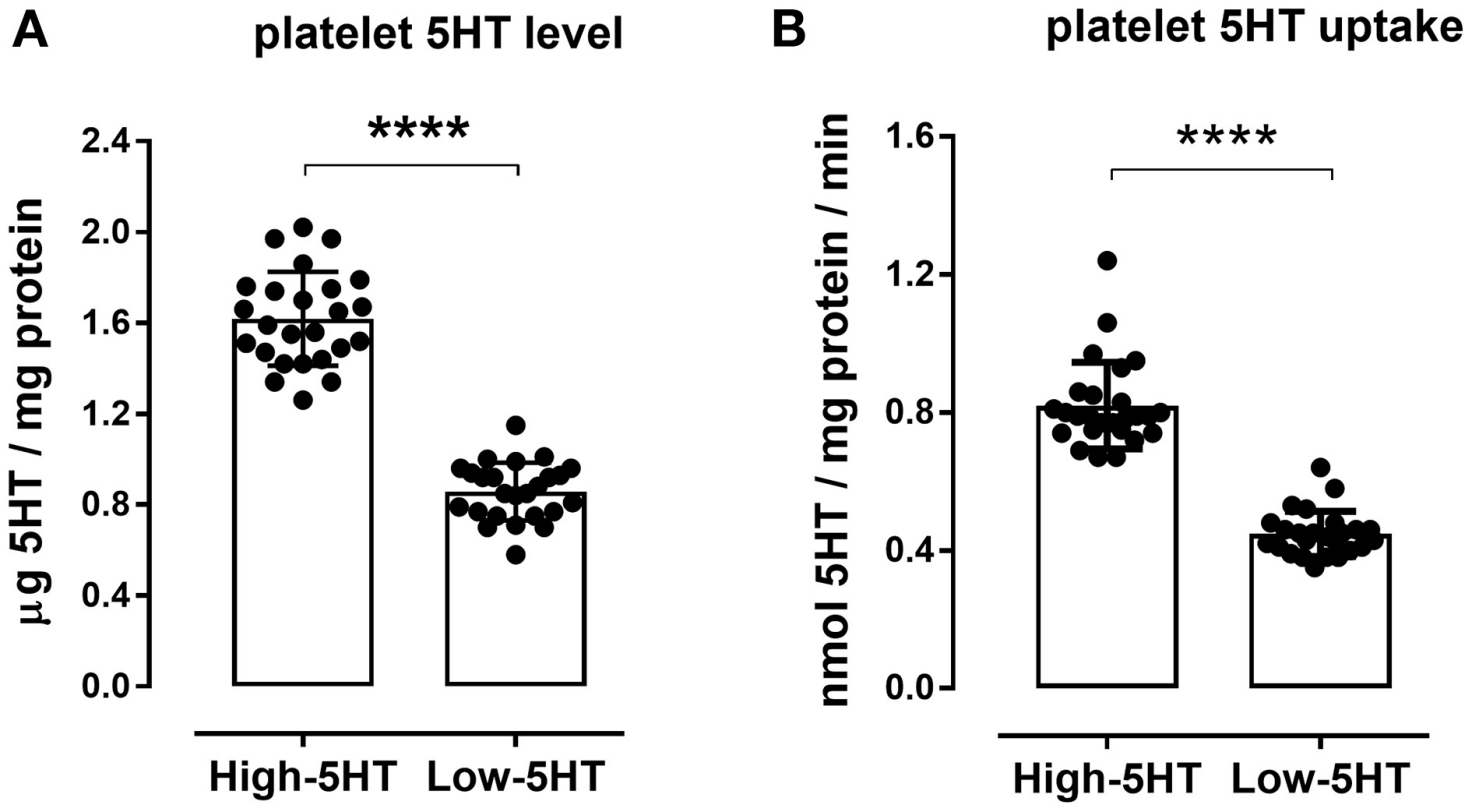
Platelet serotonin parameters in high-5HT and low-5HT animals included in the experiments. **(A)** Platelet 5HT level and **(B)** velocity of platelet 5HT uptake, relative to platelet protein levels, were measured as described in the Methods section. Individual values and bars representing means ± SD of 25 animals per subline are shown. Values of 5HT level were 1.62 ± 0.21 and 0.86 ± 0.13, and of 5HT uptake velocity were 0.82 ± 0.13 and 0.45 ± 0.06, for high-5HT and low-5HT rats, respectively. *p*-values obtained with Student *t*-test. Values of both parameters in unselected animals are intermediate to those of the sublines. ****p < 0.0001.

**Figure 3:**
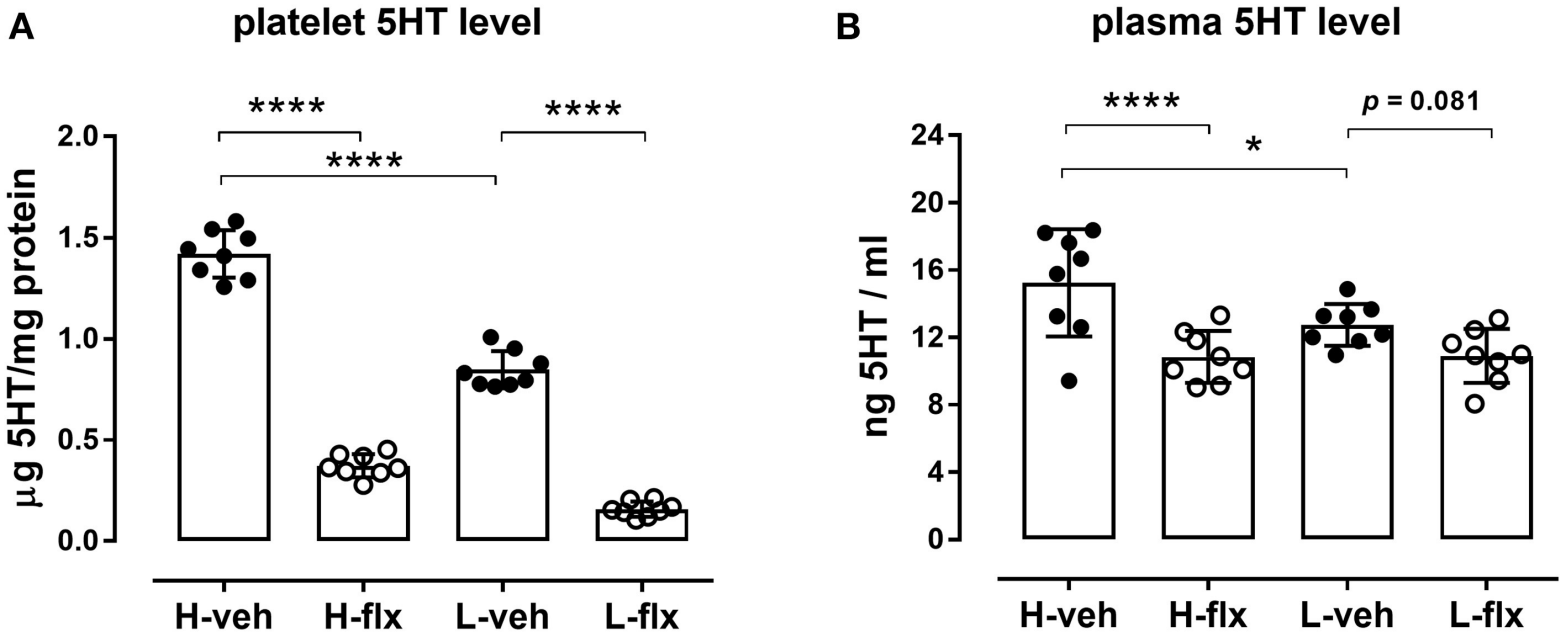
Effects of fluoxetine on **(A)** platelet 5HT levels, and **(B)** platelet-free plasma 5HT levels in animals from high-5HT and low-5HT subline (*F*_3,28_ = 373 and 8.23, *p* < 0.0001 and *p* = 0.0002, respectively, 1w-ANOVA). Blood was collected from the jugular vein after 9 days of treatment. Data are shown as individual values with group means and SD, *n* = 8 per group. *p*-values obtained by *post-hoc* (Fisher's LSD) test are given. H = high-5HT animals, L = low-5HT animals, veh = vehicle, flx = fluoxetine. *p < 0.05 and ****p < 0.0001.

Platelet 5HT parameters of the animals included in the experiments are shown in Figure 2. Rats from high-5HT and low-5HT subline displayed ~2-fold differences in platelet 5HT levels (Figure 2A) and platelet 5HT uptake velocity (Figure 2B). Fluoxetine treatment for 9 consecutive days induced a prominent reduction in platelet 5HT levels in both high-5HT and low-5HT rats (by 74 and 81%, respectively; Figure 3A). In contrast, plasma 5HT levels (Figure 3B) in rats from the two sublines were differently affected by drug treatment. Fluoxetine significantly decreased plasma 5HT levels only in animals from high-5HT subline (by 29%, *p* < 0.001) while low-5HT rats showed merely a tendency toward decreased plasma 5HT levels (15%, n.s.).

### Behavioral Measures

**Figure 4:**
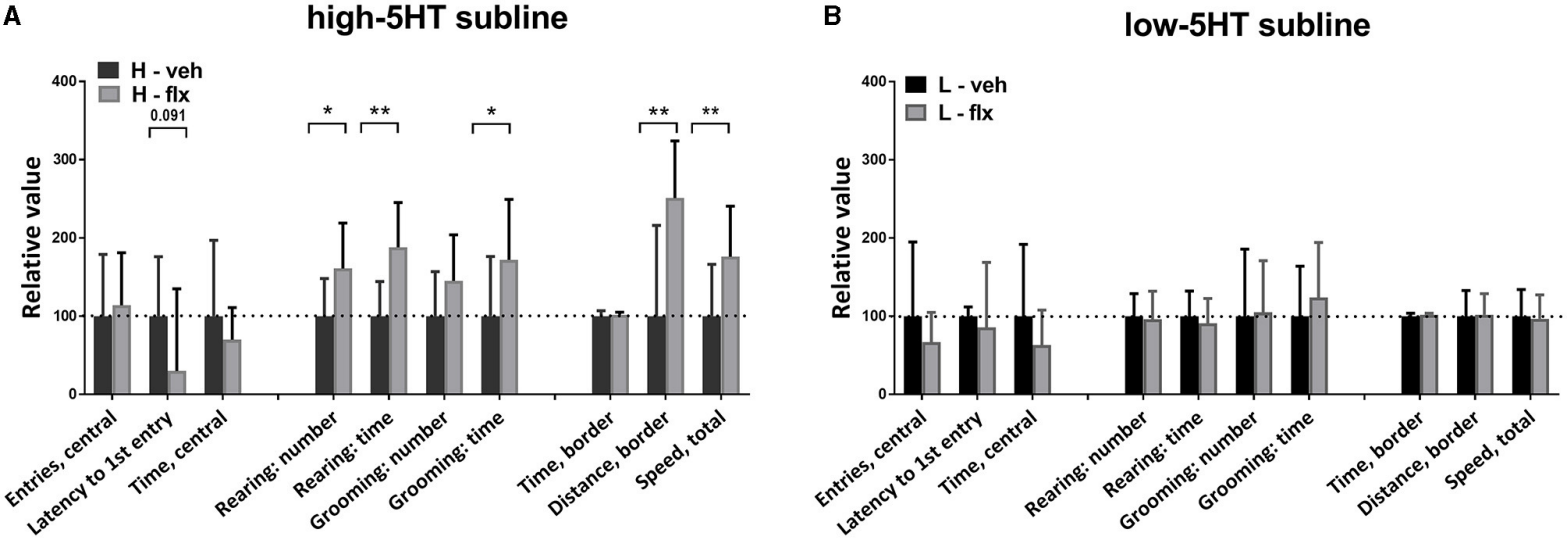
Effect of 16-day fluoxetine treatment on the behavior of **(A)** high-5HT and **(B)** low-5HT animals in the open field test. Measured behavioral categories are indicated in the X-axis legend. All values are relative to the mean/median values of the respective vehicle group (set to 100%, dotted line). Data are presented as means and SD or medians and upper quartile (Q3), *n* = 8 (vehicle-treated) or 16–17 (fluoxetine-treated). *p*-values obtained by LSD or Dunn's *post-hoc* test after 1w-ANOVA or KW test, respectively, are indicated. Full statistical data, together with absolute values of all measured parameters, are given in Supplementary Table 2. H, high-5HT animals; L, low-5HT animals; veh, vehicle; flx, fluoxetine. *p < 0.05 and **p < 0.01.

On day 17 of fluoxetine treatment, animals were subjected to behavioral testing using the open field (OF) paradigm. Figure 4 shows values of the measured behavioral parameters in the treated animals relative to the vehicle group of the same subline. The absolute values of all measured parameters and the complete statistical data are given in Supplementary Table 2. In high-5HT rats (Figure 4A), fluoxetine increased the number (by 61%, *p* = 0.015) and time (by 88%, *p* < 0.001) of rearing episodes in border zone of the OF apparatus, time (by 72%, *p* = 0.022) of grooming episodes in border zone, as well as motor activity measured as distance traveled in border zone (by 81%, *p* = 0.006) and total speed performance (by 76%, *p* = 0.004). In contrast, none of the mentioned parameters were significantly affected by fluoxetine treatment in rats from low-5HT subline (Figure 4B). The number of entries and time spent in the central zone were not altered by fluoxetine treatment in either 5HT-subline (Figure 4).

**Figure 5:**
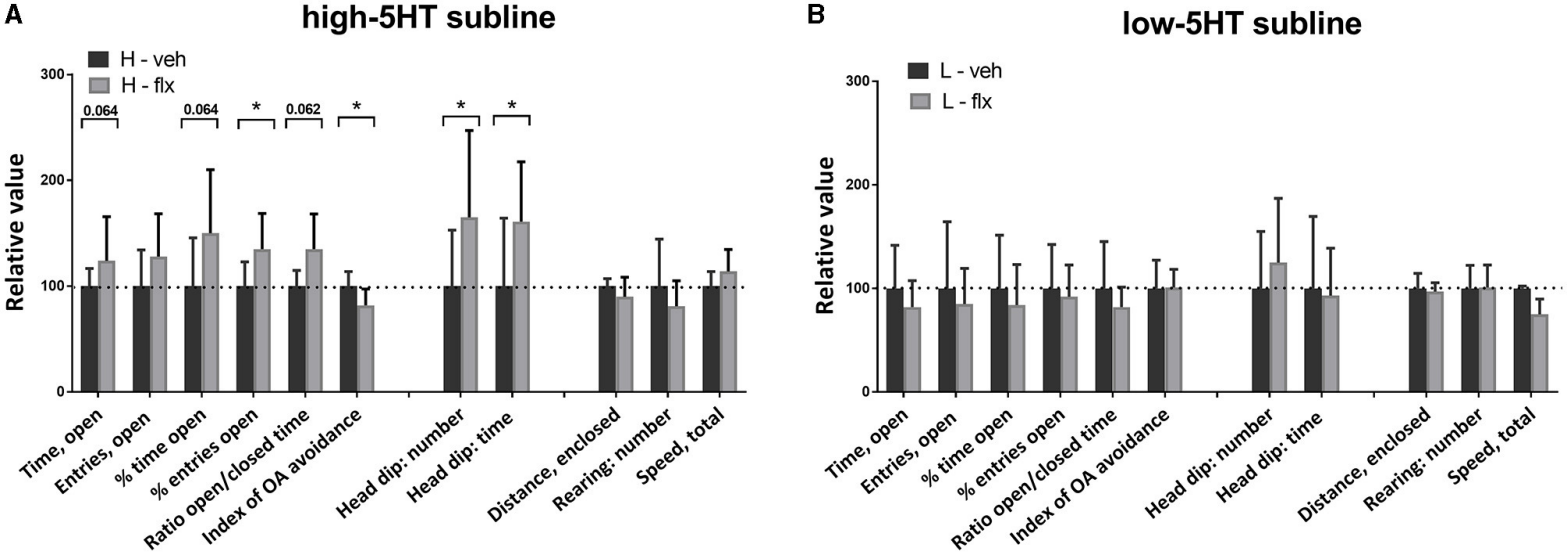
Effect of 22-day fluoxetine treatment on the behavior of **(A)** high-5HT and **(B)** low-5HT animals in the elevated plus maze test. For detailed explanation see legend to Figure 4. Complete statistical data and absolute values of all measured parameters are provided in Supplementary Table 3. *p < 0.05.

One week later (day 23 of fluoxetine treatment), we repeated the behavioral testing, this time using the elevated plus maze (EPM) paradigm. Figure 5 shows values of measured behavioral parameters in treated animals relative to the respective vehicle groups. The absolute values of all measured parameters and the complete statistical data are given in Supplementary Table 3. In high-5HT rats (Figure 5A), fluoxetine induced either significant changes or a clear trends in several anxiety-related behavioral measures. Thus, changes or trends were present in time spent in open arms (increase of 49%, *p* = 0.064), percentage of time spent in open arms (increase of 50%, *p* = 0.064), percentage of entries into open arms (increase of 35%, *p* = 0.035), ratio of open time to closed time (increase of 68%, *p* = 0.062), head dip number (increase of 49%, *p* = 0.024), head dip time (increase of 61%, *p* = 0.029), and index of open arm avoidance (decrease of 20%, *p* = 0.014), all indicating anxiolytic-like effect of fluoxetine in this subline. However, none of the aforementioned conventional or ethological EPM parameters were altered by fluoxetine treatment in low-5HT animals (Figure 5B). Parameters measuring horizontal and vertical locomotor activity in EPM test (distance traveled and rearing in enclosed arms, speed performance) were not altered by fluoxetine treatment in either 5HT subline (Figure 5). One of 17 animals in the fluoxetine-treated 5HT-low group was excluded from the statistical analyses because of almost complete immobility in both behavioral tests (outlier).

### Molecular Changes in the Frontal Cortex

**Figure 6:**
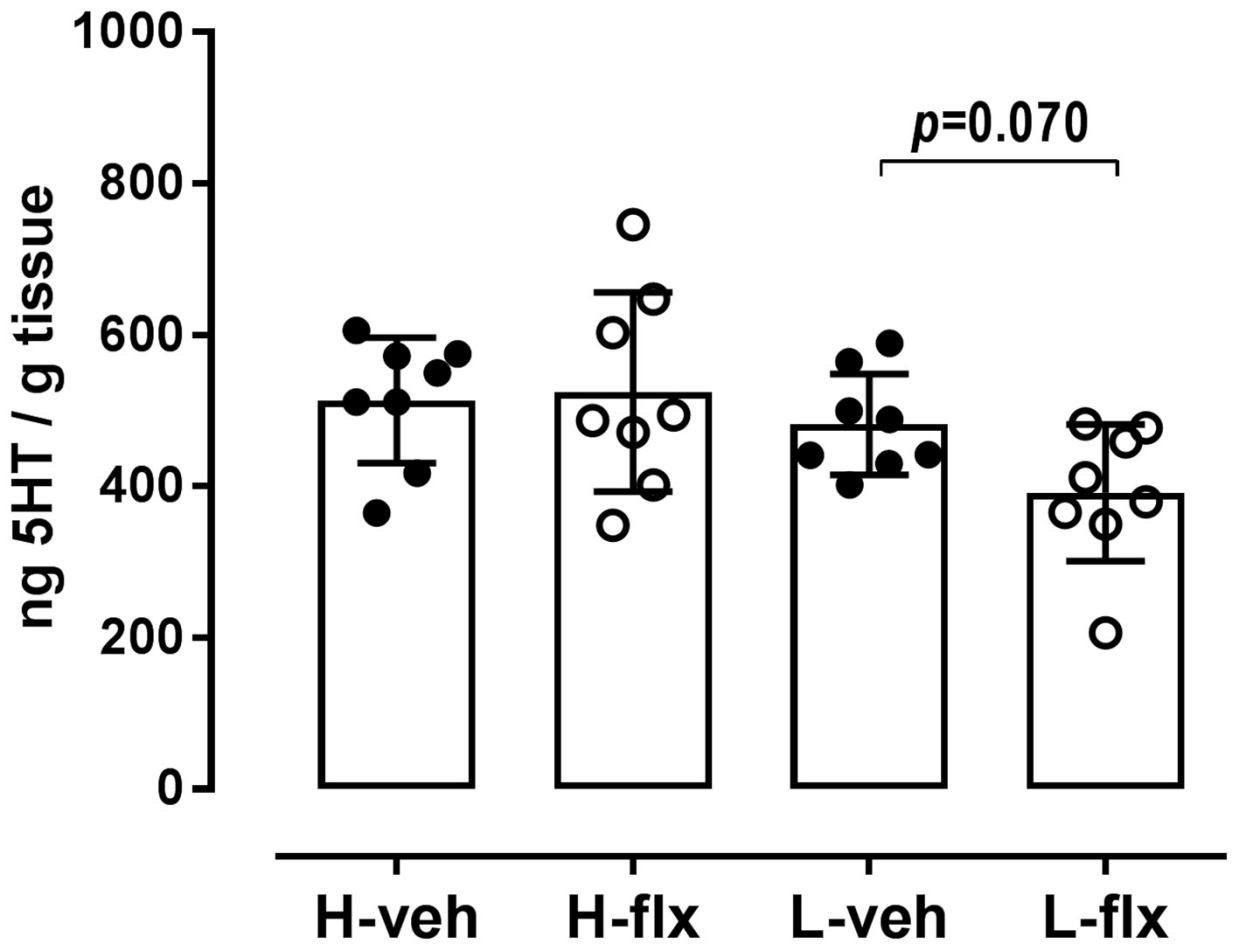
5HT content in the frontal cortex tissue. Cortical tissue was collected 48 h after the 27th injection of vehicle or fluoxetine. Data from individual animals are shown and group means ± SD are indicated, *n* = 8 per group [*F*_3,28_ = 3.173, *p* = 0.0396, 1w-ANOVA; *p*-value obtained by Fisher's LSD *post-hoc* test is given]. H, high-5HT animals; L, low-5HT animals; veh, vehicle; flx, fluoxetine.

**Figure 7:**
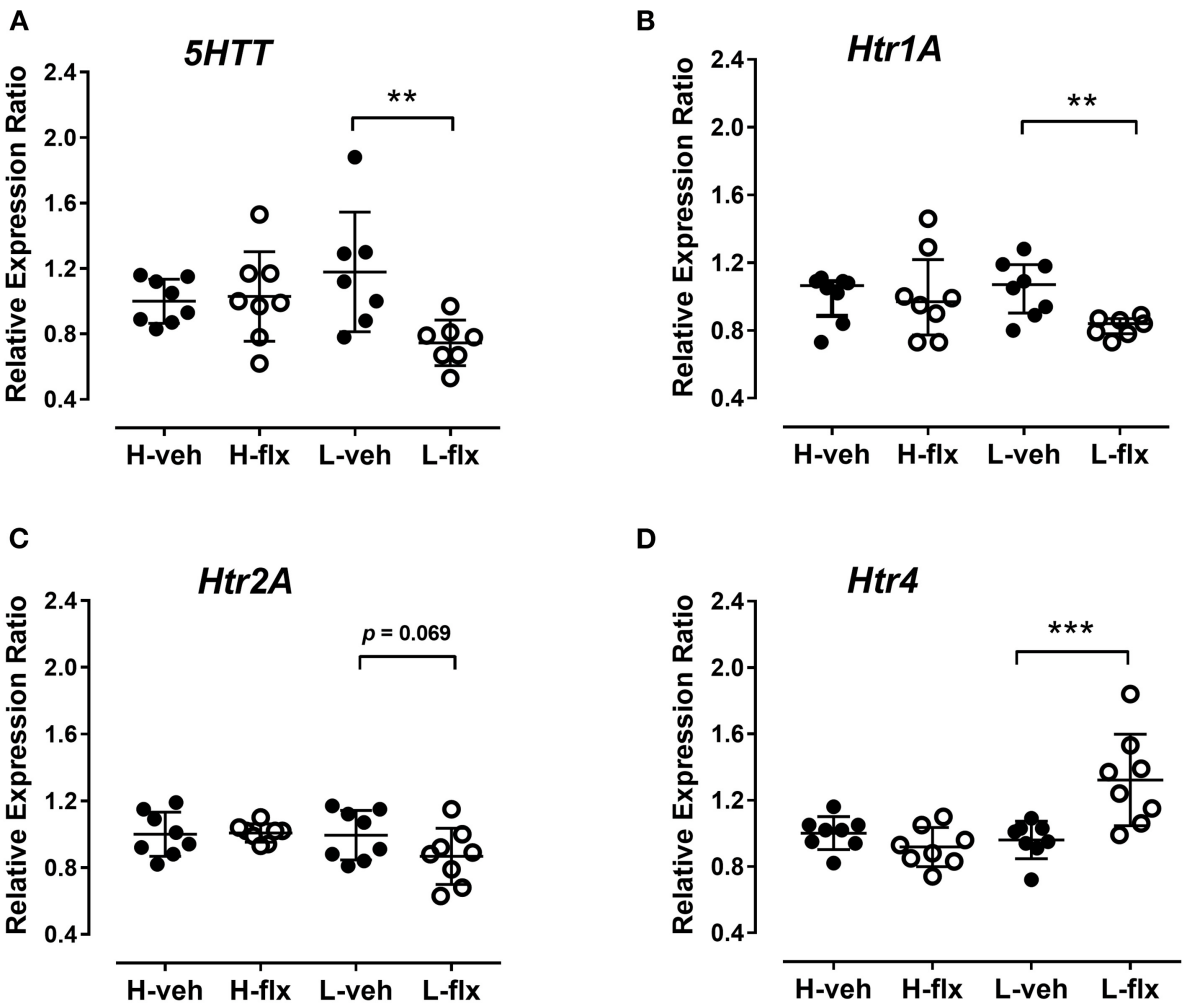
Effects of fluoxetine on cortical expression levels of serotonin-regulating genes. **(A)** Serotonin transporter [*F*_3,26_ = 3.747, *p* = 0.0232, 1w-ANOVA], **(B)** 5HT receptor subtype 1a (KW = 7.93, *p* = 0.0475, Kruskal-Wallis test), **(C)** 5HT receptor subtype 2a [*F*_3,28_ = 2.003, n.s., *p* = 0.136, 1w-ANOVA] and **(D)** 5HT receptor subtype 4 [*F*_3,28_ = 9.59, *p* = 0.0002, 1w-ANOVA]. Cortical tissue was harvested 48 h after the 27th injection of vehicle or fluoxetine. Expression levels are normalized to the mean of two reference genes, glyceraldehyde-3-phosphate dehydrogenase and actin beta. Data from individual animals are shown and group means ± SD **(A,C,D)** or group medians and IQR **(B)** are indicated (*n* = 7–8 per group). *p*-values obtained with Fisher's LSD (following 1w-ANOVA) or Dunn's test (following KW) are given. *H*, high-5HT animals; L, low-5HT animals; veh, vehicle; flx, fluoxetine. **p < 0.01 and ***p < 0.001.

After the wash-out period, animals were sacrificed, and frontal cortex samples were collected. In high-5HT rats, cortical 5HT levels were similar in the vehicle- and fluoxetine-treated groups, while in low-5HT rats, cortical 5HT levels tended to decrease (by 20%, *p* = 0.070) in fluoxetine compared to vehicle-treated group (Figure 6). Levels of serotonin transporter (*5HTT*) mRNA in the frontal cortex were also not altered by fluoxetine treatment in high-5HT subline, but were decreased (by 37%, *p* = 0.003) in low-5HT subline (Figure 7A). Moreover, high-5HT rats showed no significant changes in cortical levels of serotonin receptors type 1a (*Htr1a*), 2a (*Htr2a*), and 4 (*Htr4*) mRNAs in response to fluoxetine treatment (Figure 7). By contrast, in low-5HT rats, fluoxetine down-regulated *Htr1a* (by 22%, *p* = 0.008) and tended to decrease *Htr2a* (by 13%, *p* = 0.069), but up-regulated *Htr4* (by 38%, *p* = 0.0002) mRNA levels (Figure 7).

## Discussion

In the present study we used rat model consisting of two sublines with constitutionally different 5HT homeostasis to better understand the role of endogenous 5HT tone in modulating individual sensitivity to SSRI antidepressants. Fluoxetine was chosen as the most commonly prescribed SSRI in humans and was administered at a relatively low daily dose that could well mimic the therapeutic dose in patients. In rodents, fluoxetine plasma levels sufficient for therapeutic effect are achieved at a much lower dose than in humans and even a daily dose of 3 mg/kg increased extracellular 5HT after 2 weeks of treatment (30). The actions of fluoxetine (and other SSRIs) have been previously investigated in rodent models with genetically altered 5HT homeostasis but our study is the only one using animals with constitutionally up- and down-regulated 5HT transporter, whose activity in both sublines is still within the limits of physiological levels (18), thus likely modeling functional polymorphisms in the human *5HTT* gene.

The main finding of this study is that animals with constitutive differences in 5HT activity/homeostasis exhibit differential behavioral sensitivity to fluoxetine. Specifically, in behavioral categories measured by OF and EPM tests, chronic fluoxetine treatment induced significant changes only in animals from high-5HT subline, but not in the low-5HT subline. The anxiolytic effect in high-5HT animals was evident in the increase (significant or trend) in traditional spatio-temporal measures in the EPM test while, surprisingly, in low-5HT animals there were opposite trends. In the OF test fluoxetine treatment did not affect anxiety-like behavior in either subline. This inconsistency between the OF and EPM results might be due to differences in treatment duration prior to the behavioral testing (16 and 22 days, respectively), and/or to different settings of the respective tests, i.e., mild stress of open arena vs. higher stress triggered by EPM (31). It should be noted that the absence of a fluoxetine effect on anxiety measures in the OF test is consistent with reports that this test is not sensitive to the anxiolytic-like effect of SSRIs, but only to the effect of benzodiazepines and 5HT1A receptor agonists (32).

Regarding exploratory behavior, rearing number and time measured in the OF test were also increased only in high-5HT animals. This effect was not due to a change in mobility (number of entries into enclosed arms and distance traveled in EPM test were not affected) and is, therefore, consistent with the anxiolytic effect of fluoxetine in this subline.

Assessment of ethologically relevant measures of anxiety further supported differences in behavioral responsiveness to fluoxetine between sublines. Thus, the increased number and time of head dips in fluoxetine-treated high-5HT rats in EPM test indicated their increased tendency to actively explore the potentially dangerous area. Since head dipping is negatively correlated with anxiety (33), these data strengthen the conclusion about the anxiolytic effect of fluoxetine in high-5HT animals. It has been suggested that ethological measures are more sensitive to anxiety-modulating drugs than traditional ones (34), but in our model they showed a compliant response.

A behavioral issue that needs to be addressed is the observation that fluoxetine-induced changes in grooming behavior, similar to the behavioral categories discussed above, were present only in high-5HT animals. However, the observed increase in grooming expression is difficult to interpret in terms of anxiety. Grooming in OF/EPM tests has either been classified as a displaced behavior (35) and considered an anxiogenic response [ref. in (36)] or has been shown to represent an isolated behavioral dimension not associated with anxiety (33, 37). As for fluoxetine, its chronic administration either did not change (34) or increased self-grooming (38). It has been suggested that pharmacologically-induced grooming in rodents may be a model for obsessive-compulsive disorder (OCD) in humans (39). Since OCD has been associated with altered brain 5HT availability, and SSRIs are the mainstay of its treatment (40) our high-5HT subline may deserve further investigations in this regard.

Preclinical studies on the effect of chronic SSRIs treatment on anxiety behavior are rather contradictory, showing anxiolytic-like (about 40% studies), anxiogenic-like (20% studies) or negative outcomes [reviewed by (41)]. Our results show that individual endogenous 5HT tone may modulate direction of behavioral response to fluoxetine, and associate higher constitutional 5HT activity with the anxiolytic effect of fluoxetine. Because fluoxetine and other SSRIs are widely used to treat both depression and anxiety, future research examining the effect of SSRI treatment on depression-like behaviors in 5HT sublines is warranted. Our present results add to findings that natural variation between rodent strains in 5HT transporter binding/function is, at least in part, responsible for individual differences in SSRI sensitivity (10, 11, 42). Our results strengthen these findings as our rat sublines share the same strain background. These findings also parallel the observation that heterozygous *5HTT* mutant mice, which exhibit increased serotonergic transmission, were more sensitive to the SSRI antidepressant fluvoxamine compared to wild-type mice (43). They are also consistent with the findings in humans showing that individuals carrying the high-expressing L allele of the *5HTT* promoter polymorphism respond better to SSRIs treatment (4, 5).

Serotonin is involved in almost all physiological functions, so the differences in behavioral responsiveness to fluoxetine between our sublines could be related to various physiological processes. First, animals from 5HT sublines might have different susceptibility to stress they faced in the test apparatus – the fluoxetine-induced impairment of stress coping ability (44) may be modulated by the different stress-coping style of the 5HT sublines (shown at the first exposure to forced swimming test, personal observation). The next factor could be the difference in baseline performance in OF/EPM tests - 5HT sublines show small but consistent differences in anxiety-like behavior, i.e., higher anxiety is present in high-5HT rats (15, 16). The finding that more anxious high-5HT animals are more sensitive to chronic fluoxetine treatment is consistent with reports that antidepressants can have a profound effect in anxious patients but not in healthy individuals (45, 46), as well as with the finding that of the four mouse strains tested, only the most anxious one exhibited sensitivity to fluoxetine (47). Further, the differential sensitivity of our sublines to fluoxetine could be due to the developmental exposure to different levels of 5HT. It has been shown that pharmacologically increased 5HT activity during the prenatal/neonatal period leads to altered responsivity to SSRI treatment in adulthood (48). In 5HT sublines, the up- or down-regulation of 5HT activity is constitutional, i.e., present also during ontogeny. In this regard, the absence of behavioral effects of fluoxetine treatment in low-5HT rats is consistent with findings that congenital brain 5HT deficiency, caused by genetic manipulation of brain 5HT synthesis, reduces the efficacy of fluoxetine (49). Of note, the increase in extraneuronal 5HT concentration provoked by citalopram infusion is less pronounced in low-5HT than high-5HT rats (14).

SSRIs modulate serotonergic transmission by inhibiting the reuptake of 5HT from the extracellular space but how they achieve their antidepressant effect is still unclear. The same mechanism of SSRI action is responsible for their effect on peripheral cells, among which blood platelets are the most investigated. The true relationship between platelet/plasma and synaptic/extrasynaptic 5HT levels is uncertain, but blood 5HT parameters have long been studied as potential biomarkers of disease state or treatment response and prediction.

In our hands fluoxetine administration for 10 days significantly reduced platelet 5HT levels, as also shown in numerous experimental and clinical studies, and the extent of the reduction was similar in both sublines. In contrast, a significant reduction in platelet-free plasma 5HT levels was observed only in high-5HT rats. Long-term pharmacological blockade of platelet 5HT uptake is expected to increase 5HT concentrations in the extracellular compartment, so this decrease in plasma 5HT levels should be due to adaptive changes other than inhibition of 5HT uptake alone. Our results suggest that these adaptations may differ between 5HT sublines. Considering that plasma 5HT levels were downregulated by fluoxetine treatment only in the subline that showed anxiolytic effect of fluoxetine (5HT-high subline), it could be speculated that this parameter may be a potential early biomarker of behavioral response. A decrease of plasma 5HT levels by SSRI treatment has been found in several clinical studies (50–52), although there are also studies showing the opposite. Under basal conditions, high-5HT as compared to low-5HT animals have increased 5HT levels not only in platelets but also in platelet-free plasma [(18); Figure 2B], so their better response to fluoxetine is in line with the recent clinical study reporting that individuals with higher pretreatment plasma 5HT levels are better responders to SSRI drugs (52).

To better understand the neurobiological mechanisms underlying the differences in functional response of 5HT sublines to fluoxetine, we analyzed also 5HT levels and expression of selected 5HT-related genes in the brain frontal cortical area, which play an important role in depression and anxiety (53). Surprisingly, at the given post-treatment time-point, neurochemical and molecular changes were present only in the brains of low-5HT rats, i.e., animals that did not show behavioral response to fluoxetine. Thus, cortical 5HT concentrations tended to be decreased in fluoxetine-treated low-5HT rats while they were unchanged in high-5HT rats. Reduction of tissue 5HT level in terminal brain regions by SSRI treatment has been frequently reported (54–57), and has been shown to depend on the dose and duration of treatment (58) as well as on genetic background (55, 59).

With regard to gene expression, we focused on genes encoding *5HTT*, as a direct target of fluoxetine, and 5HT receptors *Htr1a, Htr2a*, and *Htr4*, as they have been implicated in therapeutic response to antidepressant drugs (60–62). Cortical expression levels of three of these genes were again significantly altered by fluoxetine treatment only in low-5HT, but not high-5HT, rats. Specifically, *5HTT* and *Htr1a* mRNAs were downregulated, while *Htr4* mRNA was upregulated by fluoxetine in low-5HT rats.

It is well-known that functional down-regulation of 5HTT and 5HT1A autoreceptors by repeated SSRI administration is important for the therapeutic effect. On the other hand, the effects of repeated SSRI treatment on postsynaptic 5HT1A receptors, which were also implicated in the therapeutic effect (63) are inconclusive, showing either no changes in mRNA levels (64, 65), or a reduction in mRNA with unchanged protein levels (55, 58). The indicative decrease in *Htr2a* mRNA levels in cortices from low-5HT animals suggest possible interaction of measured synaptic proteins at a given post-treatment time point. It has been suggested that fluoxetine-induced 5HT excess simultaneously activates 5HT1A and 5HT2A receptors in the pre-frontal cortex, leading to a balancing of inhibitory and excitatory responses (66). Activation of postsynaptic 5HT4 receptors was proposed to mediate therapeutic response through rapid desensitization of 5HT1A autoreceptors (62); SSRI-induced increase in extracellular 5HT availability is shown to selectively decrease 5HT4 receptor density in various 5HT projection areas (67, 68). In the pre-frontal cortex, no changes in the density of 5HT4 binding sites were found after chronic fluoxetine treatment (68), while here we observed increased mRNA expression in fluoxetine-treated low-5HT, but not high-5HT animals.

The present work has shortcomings that must be mentioned. First, behavioral tests were performed after 16 and 22 days of treatment, while gene expression levels were measured after 27 fluoxetine injections. The different timing of the respective measurements does not allow direct correlation of behavioral and molecular data. Therefore, it is difficult to interpret the results in high-5HT animals showing lack of molecular changes in the brain in the presence of behavioral response, and vice versa, the results in low-5HT animals showing lack of behavioral response in the presence of molecular changes in the brain. It is possible that the time course and/or capacity of adaptive changes necessary to produce the behavioral or molecular effect show different patterns in the 5HT-sublines. Next, the lack of protein-level measurements of the genes of interest is another limitation of our study. It is possible that measured mRNA levels may not match corresponding protein levels at the given time points. 5HTT and 5HT1A receptors internalize in both cell bodies and axon terminals after chronic fluoxetine treatment, so it is not very uncommon for (functional) radioligand binding studies and genetic expression studies to yield equivocal results (58, 69).

In conclusion, the present results show that higher constitutional 5HT activity is associated with higher sensitivity to anxiolytic effects of SSRI (fluoxetine) treatment. In addition to highlighting the role of endogenous 5HT tone as an important factor influencing behavioral response to antidepressants, our results suggest that adaptive changes in cortical *5HTT, Htr1a*, and *Htr4* expression levels in response to SSRI treatment are also dependent on endogenous 5HT tone. Further studies are needed to explain their interrelations with the behavioral response. Finally, our results support plasma 5HT level as a predictor of individual response to SSRI treatment, indicating its potential utility as an early biomarker of therapeutic response in clinical practice. In this regard, future research should include the antidepressant effects of SSRI treatment in 5HT-sublines, with low-5HT subline likely contributing to a better understanding of the neurobiology of treatment resistant anxiety/depression in humans.

## Data Availability Statement

The original contributions presented in the study are included in the article/Supplementary Material, further inquiries can be directed to the corresponding authors.

## Ethics Statement

The animal study was reviewed and approved by Bioethic Committee of the Ru$\bar{\text{d}}$er Bošković Institute, Zagreb and Bioethics Committee of the Ministry of Agriculture, Republic of Croatia.

## Author Contributions

LČ-Š and JŠ conceptualized the project. MK was the main author involved in investigation and formal analysis of data. The investigation and analysis was partly conducted by GM and BM (behavioral studies) and JŠ (molecular part). LČ-Š, JŠ, and AT were involved in supervision, validation for experiments, and provided resources. Original draft writing was performed by MK, JŠ, and LČ-Š review and editing by all authors. All authors have approved the final manuscript.

## Funding

This research was funded by Croatian Science Foundation (IP-2014-09-7827) and Croatian Ministry of Science (098-108-1870-2395) and co-financed by the Scientific Centre of Excellence for Basic, Clinical, and Translational Neuroscience (GA KK01.1.1.01.0007) funded by the European Union through the European Regional Development Fund.

## Conflict of Interest

The authors declare that the research was conducted in the absence of any commercial or financial relationships that could be construed as a potential conflict of interest.

## Publisher's Note

All claims expressed in this article are solely those of the authors and do not necessarily represent those of their affiliated organizations, or those of the publisher, the editors and the reviewers. Any product that may be evaluated in this article, or claim that may be made by its manufacturer, is not guaranteed or endorsed by the publisher.
