# Supplementary material for: Constitutive Serotonin Tone Modulates Molecular and Behavioral Response to Chronic Fluoxetine Treatment: A Study on Genetic Rat Model
Source: Front Psychiatry. 2021 Oct 1;12:741222. doi: 10.3389/fpsyt.2021.741222 (PMC8517234; doi:10.3389/fpsyt.2021.741222)
Supplement: Supplementary file 1 [file Presentation_1.pdf]

Supplementary Material

Supplementary Table S1. Gene-specific primers used in real-time PCR (qPCR) analyses.

**Name of gene**	**Symbol**	**Primer sequence (5'- 3')**	**Sequence source**
Glyceraldehyde-3-phosphate dehydrogenase	*Gapdh*	f: TGCCCCCATGTTTGTGATG<br>r: TGGTGGTGCAGGATGCATT	Abumaria et al., 2008
Actin beta	*Actb*	f: GCGCAAGTACTCTGTGTGGA<br>r: ACATCTGCTGGAAGGTGGAC	Erjavec et al., 2016
Serotonin transporter	*5HTT*	f: TCTGAAAAGCCCCACTGGACT<br>r: TAGGACCGTGTCTTCATCAGGC	Abumaria et al., 2008
5HT receptor subtype 1A	*Htr1a*	f: GTCCTGCCTTTCTGTGAAAGCA<br>r: TATGGCACCCAACAACGCA	Abumaria et al., 2008
5HT receptor subtype 2A	*Htr2a*	f: TGGTCATCATGGCAGTGTCC<br>r: CCACCGGTACCCATACAGGA	NM_017254.1
5HT receptor subtype 4	*Htr4*	f: TCTTTCAGACGTGCCTTCCT<br>r: GTGACACCGACTCTCCCATT	NM_012853.1

f, forward; r, reverse.

Abumaria, N., Ribic, A., Anacker, C., Fuchs, E., Flügge, G., (2008). Stress upregulates TPH1 but not TPH2 mRNA in the rat dorsal raphe nucleus: identification of two TPH2 mRNA splice variants. *Cell Mol. Neurobiol*. 28, 331–42. doi: 10.1007/s10571-007-9259-5.

Erjavec, I., Bordukalo-Niksic, T., Brkljacic, J., Grcevic, D., Mokrovic, G., Kesic, et al. (2016). Constitutively elevated blood serotonin is associated with bone loss and type 2 diabetes in rats. *PLoS One* 11. doi: 10.1371/journal.pone.0150102.

Supplementary Table S2. Behavioral parameters measured in an open field test after 16 days of fluoxetine treatment.

	Behavioral category	H-veh	H-flx	L-veh	L-flx	ANOVA or KW test		post-hoc test, *p*-value	
						F- or KW-value	*p*-value	H-flx *vs* H-veh	L-flx *vs* L-veh
Total	Distance travelled (m)	6,30 ± 4,11	11,0 ± 4,06	9,67 ± 3,27	9,39 ± 2,90	3,13 AN	0,035	0,004	0,859
	Speed (cm/s)	2,09 ± 1,38	3,68 ± 1,35	3,21 ± 1,10	3,13 ± 0,98	3,17 AN	0,033	0,004	0,877
Border zone	Entries (no)	7,38 ± 5,04	8,29 ± 4,25	7,13 ± 5,79	5,00 ± 2,13	1,81 AN	0,159	0,607	0,242
	Time spent (s)	280 ± 19,5	286 ± 8,28	288 ± 11,3	292 ± 5,59	2,55 AN	0,068	0,188	0,333
	Distance (m)	3,83 (5,58)	9,62 (6,15)	8,16 (3,82)	8,33 (4,27)	7,81 KW	0,050	0,006	0,956
	Rearing: number	12,9 ± 6,15	20,8 ± 7,40	22,3 ± 6,36	21,3 ± 7,95	3,04 AN	0,039	0,015	0,752
	Rearing: time (s)	18,5 ± 8,19	34,7 ± 10,6	36,8 ± 11,9	33,6 ± 11,7	5,13 AN	0,004	0,001	0,507
	Rearing: latency (s)	10,5 (22,0)	4,70 (3,80)	17,1 (8,20)	4,55 (6,70)	11,4 KW	0,010	0,037	0,010
	Grooming: number	2,75 ± 1,58	4,00 ± 1,62	2,63 ± 2,26	2,75 ± 1,73	1,96 AN	0,134	0,106	0,871
	Grooming: time (s)	14,4 ± 11,0	24,8 ± 11,1	13,5 ± 8,64	16,7 ± 9,48	3,41 AN	0,025	0,022	0,472
	Grooming: latency (s)	93,5 ± 39,3	125 ± 68,0	159 ± 119	164 ± 65,0	2,00 AN	0,128	0,327	0,869
Central zone	Entries (no)	6,38 ± 5,04	7,09 ± 4,25	6,13 ± 5,79	4,13 ± 2,31	1,64 AN	0,193	0,610	0,274
	Time spent (s)	20,2 ± 19,5	14,1 ± 8,28	12,3 ± 11,3	7,76 ± 5,59	2,55 AN	0,068	0,187	0,333
	Distance travelled (m)	0,45 (0,57)	1,10 (1,57)	0,72 (0,93)	0,63 (0,88)	8,28 KW	0,041	0,025	0,997
	Latency to 1st entry (s)	93,8 (134)	27,7 (121)	50,5 (28,8)	13,3 (44,1)	3,59 KW	0,309	0,091	0,620

Data are presented as means ± SD or median (IQR), as appropriate, n = 8 (vehicle-treated groups) or 16-17 (fluoxetine-treated groups). Statistical comparisons were performed using the one-way ANOVA (AN) or Kruskal-Wallis (KW) test as described in the Materials and Methods section. Indicated are F- or KW-values as well as *post hoc p*-values for differences between vehicle-treated and fluoxetine-treated groups, calculated using Fisher's LSD test or Dunn's test, respectively. H = high-5HT animals, L = low-5HT animals, veh = vehicle, flx = fluoxetine, no = number, m = meter, s = second

Supplementary Table S3. Behavioral parameters measured in an elevated plus maze test performed after 22 days of fluoxetine treatment.

	Behavioral category	H-veh	H-flx	L-veh	L-flx	ANOVA or KW test		post-hoc test, *p*-value	
						F- or KW-value	*p*-value	H-flx *vs* H-veh	L-flx *vs* L-veh
Total	Distance travelled (m)	9,22 (1,68)	9,88 (4,31)	13,2 (4,50)	10,3 (2,64)	7,05 KW	0,070	0,316	0,495
	Speed (cm/s)	2,90 (0,65)	3,30 (1,30)	4,40 (1,53)	3,30 (0,80)	5,44 KW	0,143	0,364	0,347
	Entries (no)	13,5 ± 4,34	13,4 ± 3,06	14,9 ± 4,05	15,4 ± 3,16	1,12 AN	0,350	0,922	0,733
Enclosed arms	Entries (no)	9,75 ± 3,49	7,81 ± 1,56	9,25 ± 1,39	10,6 ± 2,44	4,08 AN	0,012	0,054	0,179
	Distance travelled (m)	5,42 (1,03)	4,93 (1,95)	6,08 (1,58)	5,87 (1,19)	6,28 KW	0,099	0,762	0,580
	Time spent (s)	151 ± 30,4	111 ± 28,4	132 ± 37,6	124 ± 27,8	3,38 AN	0,026	0,003	0,539
	Rearing: number	15,3 ± 6,78	12,3 ± 3,67	18,0 ± 4,04	18,3 ± 3,91	5,88 AN	0,002	0,126	0,897
Open arms	Time spent (s)	57,1 ± 26,0	85,5 ± 34,3	87,6 ± 45,1	73,3 ± 34,2	1,48 AN	0,232	0,064	0,370
	Entries (no)	3,75 ± 1,28	4,81 ± 1,52	5,63 ± 3,62	4,80 ± 1,93	1,07 AN	0,371	0,250	0,375
	Latency to 1st entry (s)	47,5 (43,4)	16,3 (35,4)	6,90 (18,7)	31,1 (41,8)	2,35 KW	0,503	0,234	0,338
	Head dip: number	7,88 ± 4,91	11,8 ± 4,16	10,6 ± 7,29	11,9 ± 6,02	1,14 AN	0,344	0,106	0,584
	Head dip: time (s)	8,29 ± 5,33	13,3 ± 4,68	11,7 ± 5,79	10,9 ± 5,36	1,79 AN	0,164	0,029	0,742
	Head dip: latency (s)	54,7 (113)	23,3 (25,6)	8,70 (18,6)	36,5 (33,8)	5,72 KW	0,126	0,141	0,091
Central square	Time spent (s)	90,6 (43,3)	96,2 (18,6)	84,4 (26,2)	91,3 (17,2)	1,92 KW	0,599	0,461	0,459
	Latency to 1st exit (s)	33,1 ± 15,3	24,0 ± 17,1	37,8 ± 15,2	32,6 ± 19,5	1,41 AN	0,253	0,229	0,491
	Head dip: number	3,63 ± 1,92	6,35 ± 3,35	4,00 ± 2,20	5,19 ± 2,48	2,43 AN	0,078	0,024	0,318
	Head dip: time (s)	5,38 ± 3,58	8,56 ± 4,45	4,65 ± 2,54	7,30 ± 3,97	2,25 AN	0,096	0,082	0,125
Derived measures	% time open	19,0 ± 8,68	28,5 ± 11,4	29,2 ± 15,1	24,4 ± 11,4	1,48 AN	0,232	0,064	0,370
	% entries open	28,3 ± 6,48	38,2 ± 9,50	34,9 ± 14,8	32,2 ± 10,7	1,83 AN	0,155	0,035	0,550
	Ratio time open/closed	0,48 (0,27)	0,65 (0,31)	0,67 (0,49)	0,54 (0,34)	4,18 KW	0,242	0,062	0,433
	Index of OA avoidance	73,0 ± 10,1	59,6 ± 11,3	64,8 ± 17,7	65,7 ± 11,3	2,23 AN	0,098	0,014	0,869

Data are presented as means ± SD, or median (IQR) as appropriate n = 8 (vehicle-treated groups) or 16-17 (fluoxetine-treated groups). Statistical comparisons were performed by one-way ANOVA (^{AN}) or Kruskal-Wallis (^{KW}) test as described in the Materials and Methods section. Indicated are F- or KW-values as well as *post hoc p*-values for differences between vehicle-treated and fluoxetine-treated groups, calculated using Fisher's LSD test or Dunn's test, respectively. H = high-5HT animals, L = low-5HT animals, veh = vehicle, flx = fluoxetine, no = number, m = meter, s = second, OA = open arm.
